# Robust Exponential Memory in Hopfield Networks

**DOI:** 10.1186/s13408-017-0056-2

**Published:** 2018-01-16

**Authors:** Christopher J. Hillar, Ngoc M. Tran

**Affiliations:** 1 Redwood Center for Theoretical Neuroscience, Berkeley, CA USA; 2 University of Texas, Austin, Austin, TX USA

**Keywords:** Hopfield network, Recurrent dynamics, Exponential codes, Error-correcting, Shannon optimal, Minimum probability flow, Hidden clique

## Abstract

The Hopfield recurrent neural network is a classical auto-associative model of memory, in which collections of symmetrically coupled McCulloch–Pitts binary neurons interact to perform emergent computation. Although previous researchers have explored the potential of this network to solve combinatorial optimization problems or store reoccurring activity patterns as attractors of its deterministic dynamics, a basic open problem is to design a family of Hopfield networks with a number of noise-tolerant memories that grows exponentially with neural population size. Here, we discover such networks by minimizing probability flow, a recently proposed objective for estimating parameters in discrete maximum entropy models. By descending the gradient of the convex probability flow, our networks adapt synaptic weights to achieve robust exponential storage, even when presented with vanishingly small numbers of training patterns. In addition to providing a new set of low-density error-correcting codes that achieve Shannon’s noisy channel bound, these networks also efficiently solve a variant of the hidden clique problem in computer science, opening new avenues for real-world applications of computational models originating from biology.

## Introduction

Discovered first by Pastur and Figotin [1] as a simplified spin glass [2] in statistical physics, the Hopfield model [3] is a recurrent network of *n* linear threshold McCulloch–Pitts [4] neurons that can store $n/(4 \ln n)$ binary patterns [5] as distributed “memories” in the form of auto-associative fixed-point attractors. While several aspects of these networks appeared earlier (see, e.g., [6] for dynamics and learning), the approach nonetheless introduced ideas from physics into the theoretical study of neural computation. The Hopfield model and its variants have been studied intensely in theoretical neuroscience and statistical physics [7], but investigations into its utility for memory and coding have mainly focused on storing collections of patterns *X* using a “one-shot” *outer-product rule* (OPR) for learning, which essentially assigns abstract synaptic weights between neurons to be their correlation, an early idea in neuroscience [8, 9]. Independent of learning, at most 2*n* randomly generated dense patterns can be simultaneously stored in networks with *n* neurons [10].

Despite this restriction, super-linear capacity in Hopfield networks is possible for special pattern classes and connectivity structures. For instance, if patterns to memorize contain many zeros, it is possible to store nearly a quadratic number [11]. Other examples are random networks, which have ${\approx}1.22^{n}$ attractors asymptotically [12], and networks storing all permutations [13]. In both examples of exponential storage, however, memories have vanishingly small basins of attraction, making them ill-suited for noise-tolerant pattern storage. Interestingly, the situation is even worse for networks storing permutations: any Hopfield network storing permutations will not recover the derangements (more than a third of all permutations) from asymptotically vanishing noise (see Theorem 4, proved in Sect. 5).

In this note, we design a family of sparsely connected *n*-node Hopfield networks with (asymptotically, as $n \to\infty$)

1 $$ {\sim} \frac{2^{\sqrt{2n} + \frac{1}{4}}}{n^{1/4} \sqrt{\pi}} $$

robustly stored fixed-point attractors by minimizing “probability flow” [14, 15]. To our knowledge, this is the first rigorous demonstration of super-polynomial noise-tolerant storage in recurrent networks of simple linear threshold elements. The approach also provides a normative, convex, biologically plausible learning mechanism for discovering these networks from small amounts of data and reveals new connections between binary McCulloch–Pitts neural networks, efficient error-correcting codes, and computational graph theory.

## Background

The underlying probabilistic model of data in the Hopfield network is the non-ferromagnetic *Lenz–Ising model* [16] from statistical physics, more generally called a Markov random field in the literature, and the model distribution in a fully observable Boltzmann machine [17] from artificial intelligence. The states of this discrete distribution are length *n* binary column vectors ${\mathbf {x}} = (x_{1},\ldots, x_{n}) \in\{0,1\}^{n}$ each having probability $p_{{\mathbf {x}}} := \frac{1}{Z} \exp ( - E_{\mathbf{x}} )$, in which $E_{\mathbf {x}} := -\frac{1}{2}\mathbf {x}^{\top} \mathbf {W} \mathbf {x} + \theta^{\top}\mathbf {x}$ is the *energy* of a state, **W** is an *n*-by-*n* real symmetric matrix with zero diagonal (the *weight matrix*), the vector $\theta\in\mathbb {R}^{n}$ is a *threshold* term, and $Z := \sum_{\mathbf{x}}\exp(-E_{\mathbf {x}})$ is the *partition function*, the normalizing factor ensuring that $p_{\mathbf{x}}$ represents a probability distribution. In theoretical neuroscience, rows $\mathbf{W}_{e}$ of the matrix **W** are interpreted as abstract “synaptic” weights $W_{ef}$ connecting neuron *e* to other neurons *f*.

**Fig. 1:**
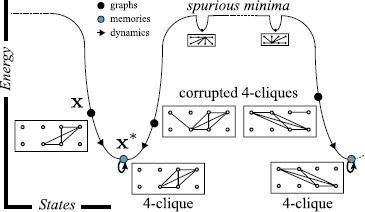
Energy landscape and discrete dynamics in a Hopfield network having robust storage of all 4-cliques in graphs on 8 vertices. The deterministic network dynamics sends three corrupted cliques to graphs with smaller energy, converging on the underlying 4-clique attractors

The pair $(\mathbf{W}, \theta)$ determines an asynchronous deterministic (“zero-temperature”) *dynamics* on states **x** by replacing each $x_{e}$ in **x** with the value:

2 $$ x_{e} = \textstyle\begin{cases} 1 & \text{if } \sum_{f \neq e} {W_{ef} x_{f}} > \theta_{e}, \\0 & \text{otherwise}, \end{cases} $$

in a (usually initialized randomly) fixed order through all neurons $e = 1, \ldots, n$. The quantity $I_{e} := \langle\mathbf{W}_{e}, \mathbf{x} \rangle$ in (2) is often called the *feedforward input* to neuron *e* and may be computed by linearly combining input signals from neurons with connections to *e*. Let $\Delta E_{e}$ (resp. $\Delta x_{e} = \pm1, 0$) be the energy (resp. bit) change when applying (2) at neuron *e*. The relationship

3 $$ \Delta E_{e} = -\Delta x_{e} (I_{e} - \theta_{e}) $$

guarantees that network dynamics does not increase energy. Thus, each initial state **x** will converge in a finite number of steps to its *attractor* $\mathbf {x}^{*}$ (also called in the literature *fixed-point*, *memory*, or *metastable state*); e.g., see Fig. 1. The biological plausibility and potential computational power [18] of the dynamics update (2) inspired both early computer [19] and neural network architectures [4, 20].

We next formalize the notion of robust fixed-point attractor storage for families of Hopfield networks. For $p \in[0,\frac{1}{2}]$, the *p-corruption* of **x** is the random pattern $\mathbf {x}_{p}$ obtained by replacing each $x_{e}$ by $1-x_{e}$ with probability *p*, independently. The *p*-corruption of a state differs from the original by *pn* bit flips on average so that for larger *p* it is more difficult to recover the original binary pattern; in particular, $\mathbf{x}_{\frac{1}{2}}$ is the uniform distribution on $\{0,1\}^{n}$ (and thus independent of **x**). Given a Hopfield network, the attractor $\mathbf{x}^{\ast}$ has $(1-\varepsilon )$*-tolerance* for a *p*-corruption if the dynamics can recover $\mathbf{x}^{\ast}$ from $(\mathbf{x}^{\ast})_{p}$ with probability at least $1-\varepsilon $. The *α-robustness* $\alpha(X, \varepsilon )$ for a set of states *X* is the most *p*-corruption every state $(1-\varepsilon )$-tolerates.

At last, we say that a sequence of Hopfield networks $\mathcal{H}_{n}$ *robustly stores* states $X_{n}$ with robustness index $\alpha> 0$ if the following limit exists and equals the number *α*:

4 $$ \lim_{\varepsilon \to0^{+}} \lim_{n \to\infty} \inf \bigl\{ \alpha (X_{n},\varepsilon ), \alpha(X_{n+1},\varepsilon ), \ldots \bigr\} = \alpha. $$

If *α* is the robustness index of a family of networks, then the chance that dynamics does not recover an *α*-corrupted memory can be made as small as desired by devoting more neurons. (Note that by definition, we always have $\alpha\leq1/2$.)

To determine parameters $(\mathbf{W}, \theta)$ in our networks from a set of training patterns $X \subseteq\{0,1\}^{n}$, we minimize the following *probability flow* objective function [14, 15]:

5 $$ \frac{1}{|X|} \sum_{\mathbf{x} \in X} \sum _{\mathbf{x}' \in\mathcal {N}(\mathbf{x})} \exp \biggl(\frac{E_{\mathbf{x}}-E_{\mathbf {x}'}}{2} \biggr), $$

in which $\mathcal{N}(\mathbf{x})$ are those neighboring states $\mathbf {x}'$ differing from **x** by a single flipped bit. It is elementary that a Hopfield network has attractors *X* if and only if the probability flow (5) can be arbitrarily close to zero, motivating the application of minimizing (5) to find such networks [15]. Importantly, the probability flow is a convex function of the parameters, consists of a number of terms linear in *n* and the size of *X*, and avoids the exponentially large partition function *Z*. We remark that the factor of $\frac{1}{2}$ inside of the exponential in (5) will turn out to be unimportant for our analysis; however, we keep it to be consistent with the previous literature on interpreting (5) as a probability density estimation objective.

Let *v* be a positive integer and set $n = \frac{v(v-1)}{2}$. A state **x** in a Hopfield network on *n* nodes represents a simple undirected graph *G* on *v* vertices by interpreting a binary entry $x_{e}$ in **x** as indicating whether edge *e* is in *G* ($x_{e} = 1$) or not ($x_{e} = 0$). A *k*-*clique* **x** is one of the ${v \choose k} = \frac{v \cdot(v-1)\cdots(v-k+1)}{k \cdot(k-1)\cdots2 \cdot1}$ graphs consisting of *k* fully connected nodes and $v-k$ other isolated nodes. Below, in Sect. 3, we will design Hopfield networks that have all *k*-cliques on 2*k* (or $2k-2$) vertices as robustly stored memories. For large *n*, the count ${2k \choose k}$ approaches (1) by Stirling’s approximation. Figure 1 depicts a network with $n = 28$ neurons storing 4-cliques in graphs on $v = 8$ vertices.

## Results

**Fig. 2:**
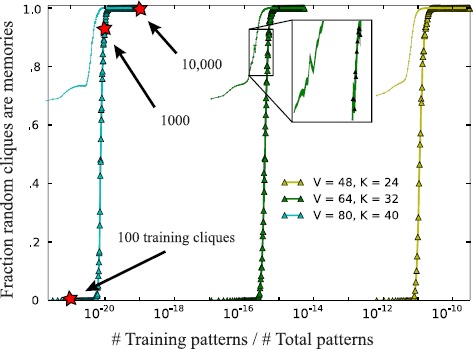
Learning critical networks with exponential memory by minimizing probability flow on few training patterns. For numbers of vertices $v = 80, 64, 48$ ($k = 40, 32, 24$) with 50 trials each, the average percent of 1000 randomly drawn cliques that are memories vs. the fraction of training samples to total number of *k*-cliques. Inset displays enlarged version of the region demarcated by *black square*; *filled regions* indicate standard deviation errors over these 50 trials. *Dotted lines* are average percentage of correct bits after converging dynamics

Our first result is that numerical minimization of probability flow over a vanishingly small critical number of training cliques determines linear threshold networks with exponential attractor memory. We fit all-to-all connected networks on $n = 3160, 2016, 1128$ neurons ($v = 80, 64, 48$; $k=40,32, 24$) with increasing numbers of randomly generated *k*-cliques as training data *X* by minimizing (5) with the limited-memory Broyden–Fletcher–Goldfarb–Shanno (L-BFGS) algorithm [21] (implemented in the programming language Python’s package SciPy). In Fig. 2, we plot the percentage of 1000 random new *k*-cliques that are fixed-points in these networks after training as a function of the ratio of training set size to total number of *k*-cliques. Each triangle in the figure represents the average of this fraction over 50 networks, each given the same number of randomly generated (but different) training data. The finding is that a critical number of training samples allows for storage of all *k*-cliques. Moreover, this count is significantly smaller than the total number of patterns to be learned.

**Fig. 3:**
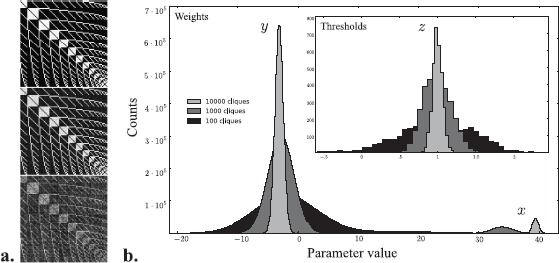
Distribution of network parameters learned by minimizing probability flow (MPF) sharpens around three critical values. **(a)** Portion of network weights **W** after minimizing (5) given 100 (*bottom*), 1000 (*middle*), or 10,000 (*top*) random 40-cliques *X* (of about 10^23^ in total) on $v = 80$ vertices. These networks represent the marked points in Fig. 2. **(b)** Histograms of weight and threshold parameters for networks in (a) (histogram of thresholds *θ* in inset). Network parameters are scaled so that thresholds have mean 1 (this does not affect the dynamics). Groups of similar network weights and thresholds are labeled with corresponding parameter *x*, *y*, *z*

In Fig. 3(a), we display a portion of the weight matrix with minimum probability flow representing a $v = 80$ network (4,994,380 weight and threshold parameters) given 100 (${\approx}1\mathrm{e}{-}21\%$ of all 40-cliques), 1000 ($1\mathrm{e}{-}20\%$), or $10\text{,}000$ ($1\mathrm{e}{-}19\%$) randomly generated 40-cliques as training data; these are the three special starred points in Fig. 2. In Fig. 3(b), we also plot histograms of learned parameters from networks trained on data with these three sample sizes. The finding is that weights and thresholds become highly peaked and symmetric about three limiting quantities as sample size increases.

We next analytically minimize probability flow to determine explicit networks achieving robust exponential storage. To simplify matters, we first observe by a symmetrizing argument (see Sect. 5) that there is a network storing all *k*-cliques if and only if there is one with constant threshold $\theta = (z, \ldots, z) \in\mathbb{R}^{n}$ and satisfying for each pair $e \neq f$, ether $W_{ef} = x$ (whenever *e* and *f* share one vertex) or $W_{ef} = y$ (when *e* and *f* are disjoint). Weight matrices approximating this symmetry can be seen in Fig. 3(a). (Note that this symmetry structure on the weights is independent of clique size *k*.) In this case, the energy of a graph *G* with $\#E(G)$ edges is the following linear function of $(x,y,z) \in\mathbb {R}^{3}$:

6 $$ E_{G}(x,y,z) = - x \cdot S_{1}(G) - y \cdot S_{0}(G) + z \cdot\#E(G), $$

in which $S_{1}(G)$ and $S_{0}(G)$ are the number of edge pairs in the graph *G* with exactly one or zero shared vertices, respectively.

Consider the minimization of (5) over a training set *X* consisting of all ${v \choose k}$ *k*-cliques on $v = 2k-2$ vertices (this simplifies the mathematics), restricting networks to our 3-parameter family $(x,y,z)$. When $y = 0$, these networks are sparsely connected, having a vanishing number of connections between neurons relative to total population size. Using single variable calculus and Eq. (6), one can check that, for any fixed positive threshold *z*, the minimum value of (5) is achieved uniquely at the parameter setting $(x,0,z)$, where

7 $$ x = \frac{2z}{3k - 5}. $$

This elementary calculation gives our first main theoretical contribution.

### Theorem 1

*McCulloch–Pitts attractor networks minimizing probability flow can achieve robust exponential pattern storage*.

We prove Theorem 1 using the following large deviation theory argument; this approach also allows us to design networks achieving optimal robustness index $\alpha= 1/2$ (Theorem 2). Fix $v = 2k$ (or $v = 2k-2$) and consider a *p*-corrupted clique. Using Bernstein’s concentration inequality for sums of Bernoulli binary random variables [22] (“coin flips”), it can be shown that with high probability (i.e., approaching 1 as $v \to\infty$) an edge in the clique has 2*k* neighboring edges at least, on average (see Corollary 1).

This gives the fixed-point requirement from (2):

$$ 2kx + o(x\sqrt{k}\ln k) > z. $$

On the other hand, a non-clique edge sharing a vertex with the clique has $k(1+2p)$ neighbors at most, on average. Therefore, for a *k*-clique to be a robust fixed-point, this forces again from (2):

$$ k(1+2p)x + o(x\sqrt{k}\ln k) \leq z, $$

and any other edges will disappear when this holds. ($o(\cdot)$ is “little-o” notation.)

It follows that the optimal setting (7) for *x* minimizing probability flow gives robust storage (with a single parallel dynamics update) of all *k*-cliques for $p < 1/4$. This proves Theorem 1 (see Sect. 5 for the full mathematical details).

It is possible to do better than robustness index $\alpha= 1/4$ by setting $x = \frac{1}{2} [\frac{z}{2k} + \frac{z}{k(1+2p)} ] = \frac{z(3+2p)}{4k(1 + 2p)}$, which satisfies the above fixed-point requirements with probability approaching 1 for any fixed $p < 1/2$ and increasing *k*. We have thus also demonstrated:

### Theorem 2

*There is a family of Hopfield networks on* $n = {2k \choose 2}$ *nodes that robustly store* ${2k \choose k} \sim\frac{2^{\sqrt{2n} + \frac {1}{4}}}{n^{1/4} \sqrt{\pi}}$ *binary patterns with maximal robustness index* $\alpha= 1/2$.

**Fig. 4:**
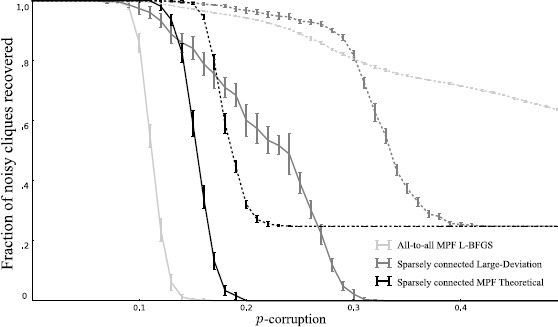
Robust exponential storage in networks of McCulloch–Pitts neurons. Error-correction performance of Hopfield networks storing all 64-cliques in $v=128$ vertex graphs using a fully connected 8128-bit network minimizing probability flow (5) on $50\text{,}000$ random 64-cliques (*light gray line*), a sparsely connected $(x, 0, 1)$ network with large deviation setting $x = \frac{3+2p}{4k(1 + 2p)}$ and $p=1/4$ (*gray*), or a sparsely connected MPF theoretical optimum (7) (*black*). Over 10 trials, 100 64-cliques chosen uniformly at random were *p*-corrupted for different *p* and then dynamics were converged initialized at noisy cliques. The plot shows the fraction of cliques completely recovered vs. pattern corruption *p* (standard deviation error bars). *Dotted lines* are average number of bits in a pattern retrieved correctly after converging network dynamics

In Fig. 4, we show robust storage of the (≈10^37^) 64-cliques in graphs on 128 vertices using three $(x,y,z)$ parameter specializations designed here.

A natural question is whether we can store a range of cliques using the same architecture. In fact, we show here that there is a network storing nearly all cliques.

### Theorem 3

*For large* *v*, *there is a Hopfield network on* $n = {v \choose 2}$ *nodes that stores all* ${\sim}2^{v}(1 - e^{-Cv})$ *cliques of size* *k* *as fixed*-*points*, *where* *k* *is in the range*:

$$m = \frac{1}{D} v \leq k \leq v = M, $$

*for constants* $C \approx0.43$, $D \approx13.93$. *Moreover*, *this is the largest possible range of* *k* *for any such Hopfield network*.

Our next result demonstrates that even robustness to vanishingly small amounts of noise is nontrivial (see Sect. 5.5 for the proof).

### Theorem 4

*Hopfield–Platt networks storing all permutations will not robustly store derangements* (*permutations without fixed*-*points*).

As a final application to biologically plausible learning theory, we derive a synaptic update rule for adapting weights and thresholds in these networks. Given a training pattern **x**, the *minimum probability flow* (MPF) learning rule moves weights and thresholds in the direction of steepest descent of the probability flow objective function (5) evaluated at $X = \{\mathbf{x}\}$. Specifically, for $e \neq f$ the rule takes the form:

8 $$ \begin{aligned} \Delta W_{ef} & \propto- x_{f} \Delta x_{e} \exp(-\Delta E_{e}/2), \\ \Delta\theta_{e} & \propto\Delta x_{e} \exp(-\Delta E_{e}/2). \end{aligned} $$

After learning, the weights between neurons *e* and *f* are symmetrized to $\frac{1}{2}(W_{ef} + W_{fe})$, which preserves the energy function and guarantees that dynamics terminates in fixed-point attractors. As update directions (8) descend the gradient of an infinitely differentiable convex function, learning rules based on them have good convergence rates [23].

Let us examine the (symmetrized) learning rule (8) more closely. Suppose first that $x_{e} = 0$ so that $\Delta x_{e} = 0$ or 1 (depending on the sign of $I_{e} - \theta_{e}$). When $\Delta x_{e} = 0$, weight $W_{ef}$ does not change; on the other hand, when $\Delta x_{e} = 1$, the weight decreases if $x_{f} = 1$ (and stays the same, otherwise). If instead $x_{e} = 1$, then $W_{ef}$ changes only if $\Delta x_{e} = -1$ or $\Delta x_{f} = -1$, in which case the update is positive when at least one of $x_{e}$, $x_{f}$ is 1 (and zero, otherwise). In particular, either (i) weights do not change (when the pattern is memorized or there is no neural activity) or (ii) when neurons *e* and *f* are both active in (8), weights increase, while when they are different, they decrease, consistent with Hebb’s postulate [9], a basic hypothesis about neural synaptic plasticity. In fact, approximating the exponential function with unity in (8) gives a variant of classical outer-product rule (OPR) learning. Note also that adaptation (8) is *local* in that updating weights between 2 neurons only requires their current state/threshold and feedforward input from nearby active neurons.

## Discussion

The biologically inspired networks introduced in this work constitute a new nonlinear error-correcting scheme that is simple to implement, parallelizable, and achieves the most asymptotic error tolerance possible [24] for low-density codes over a binary symmetric channel ($\alpha= 1/2$ in definition (4)). There have been several other approaches to optimal error-correcting codes derived from a statistical physics perspective; for a comprehensive account, we refer the reader to [25]. See also [26–29] for related work on neural architectures with large memory. Additionally, for a recent review of memory principles in computational neuroscience theory more broadly, we refer the reader to the extensive high level summary [30].

Although we have focused on minimizing probability flow to learn parameters in our discrete neural networks, several other strategies exist. For instance, one could maximize the (Bayesian) likelihood of cliques given network parameters, though any strategy involving a partition function over graphs might run into challenging algorithmic complexity issues [31]. Contrastive divergence [17] is another popular method to estimate parameters in discrete maximum entropy models. While this approach avoids the partition function, it requires a nontrivial sampling procedure that precludes exact determination of optimal parameters.

Early work in the theory of neural computation put forward a framework for neurally plausible computation of (combinatorial) optimization tasks [32]. Here, we add another task to this list by interpreting error-correction by a recurrent neural network in the language of computational graph theory. A basic challenge in this field is to design efficient algorithms that recover structures imperfectly hidden inside of others; in the case of finding fully connected subgraphs, this is called the “Hidden clique problem” [33]. The essential goal of this task is to find a single clique that has been planted in a graph by adding (or removing) edges at random.

**Fig. 5:**
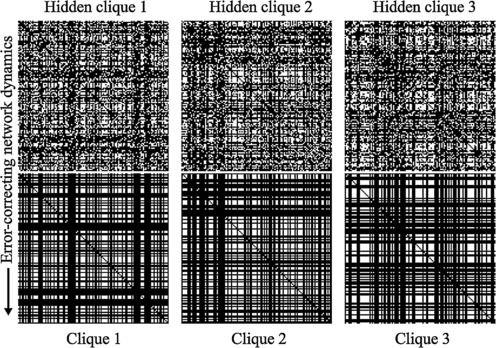
Learning to solve ≈10^37^ “Hidden clique” problems. (*Bottom*) Adjacency matrices of three 64-cliques on $v = 128$ vertices. (*Top*) Adjacency matrices of noisy versions of the cliques having, on average, 1219 bits corrupted out of $n = 8128$ from the original. Converging dynamics of a symmetric 3-parameter network $(x, y, z) = (0.0107, 0, 1)$ with minimum probability flow initialized at these noisy cliques uncovers the originals

Phrased in this language, we have discovered discrete recurrent neural networks that learn to use their cooperative McCulloch–Pitts dynamics to solve hidden clique problems efficiently. For example, in Fig. 5 we show the adjacency matrices of three corrupted 64-cliques on $v=128$ vertices returning to their original configuration by one iteration of the network dynamics through all neurons. As a practical matter, it is possible to use networks robustly storing *k*-cliques for detecting highly connected subgraphs with about *k* neighbors in large graphs. In this case, error-correction serves as a synchrony finder with free parameter *k*, similar to how “*K*-means” is a standard unsupervised approach to decompose data into *K* clusters.

In the direction of applications to basic neuroscience, we comment that it has been proposed that co-activation of groups of neurons—that is, synchronizing them—is a design principle in the brain (see, e.g., [34–36]). If this were true, then perhaps the networks designed here can help discover this phenomenon from spike data. Moreover, our networks also then provide an abstract model for how such coordination might be implemented, sustained, and error-corrected in nervous tissue.

As a final technical remark about our networks, note that our synapses are actually discrete since the probability flow is minimized at a synaptic ratio equaling a rational number. Thus, our work adds to the literature on the capacity of neural networks with discrete synapses (see, e.g., [26, 37–40]), all of which build upon early classical work with associative memory systems (see, e.g., [20, 41]).

## Mathematical Details

We provide the remaining details for the proofs of mathematical statements appearing earlier in the text.

### Symmetric 3-Parameter $(x,y,z)$ Networks

The first step of our construction is to exploit symmetry in the following set of linear inequalities:

9 $$ E_{\mathbf{c}} - E_{\mathbf{c'}} < 0, $$

where **c** runs over *k*-cliques and $\mathbf{c}'$ over vectors differing from **c** by a single bit flip. The space of solutions to (9) is the convex polyhedral cone of networks having each clique as a strict local minimum of the energy function, and thus a fixed-point of the dynamics.

The permutations $P \in P_{V}$ of the vertices *V* act on a network by permuting the rows/columns of the weight matrix ($\mathbf{W} \mapsto P \mathbf{W}P^{\top}$) and thresholds ($\theta\mapsto P \theta$), and this action on a network satisfying property (9) preserves that property. Consider the average $(\mathbf{\overline{W}}, \bar{\theta})$ of a network over the group $P_{V}$: $\mathbf{\overline {W}} := \frac{1}{v!}\sum_{P \in P_{V}}P \mathbf{W} P^{\top}$, $\bar{\theta } := \frac{1}{v!}\sum_{P \in P_{V}}P \theta$, and note that if $(\mathbf {W}, \theta)$ satisfies (9) then so does the highly symmetric object $(\mathbf{\overline{W}}, \bar{\theta})$. To characterize $(\mathbf{\overline{W}}, \bar{\theta})$, observe that $P \mathbf{\overline{W}} P^{\top}= \mathbf{\overline{W}}$ and $P \bar {\theta} = \bar{\theta}$ for all $P \in P_{V}$.

These strong symmetries imply there are *x*, *y*, *z* such that $\bar{\theta} = (z, \ldots, z) \in\mathbb{R}^{n}$ and for each pair $e \neq f$ of all possible edges:

$$ \overline{W}_{ef} = \textstyle\begin{cases} x & \mbox{if } |e \cap f| = 1, \\ y & \mbox{if } |e \cap f| = 0, \end{cases} $$

where $|e \cap f|$ is the number of vertices that *e* and *f* share.

Our next demonstration is an exact setting for weights in these Hopfield networks.

### Exponential Storage

For an integer $r \geq0$, we say that state $\mathbf{x}^{\ast}$ is *r-stable* if it is an attractor for all states with Hamming distance at most *r* from $\mathbf{x}^{\ast}$. Thus, if a state $\mathbf{x}^{\ast}$ is *r*-stably stored, the network is guaranteed to converge to $\mathbf {x}^{\ast}$ when exposed to any corrupted version not more than *r* bit flips away.

For positive integers *k* and *r*, is there a Hopfield network on $n = \binom{2k}{2}$ nodes storing all *k*-cliques *r*-stably? We necessarily have $r \leq\lfloor k/2 \rfloor$, since $2(\lfloor k/2 \rfloor+1)$ is greater than or equal to the Hamming distance between two *k*-cliques that share a $(k-1)$-subclique. In fact, for any $k > 3$, this upper bound is achievable by a sparsely connected three-parameter network.

#### Lemma 1

*There exists a family of three*-*parameter Hopfield networks with* $z = 1$, $y = 0$ *storing all* *k*-*cliques as* $\lfloor k/2 \rfloor$-*stable states*.

The proof relies on the following lemma, which gives the precise condition for the three-parameter Hopfield network to store *k*-cliques as *r*-stable states for fixed *r*.

#### Lemma 2

*Fix* $k > 3$ *and* $0 \leq r < k$. *The Hopfield network* $(\mathbf{W}(x,y), \theta(z))$ *stores all* *k*-*cliques as* *r*-*stable states if and only if the parameters* $x,y,z \in\mathbb {R}$ *satisfy*

$$ M \cdot \left [ \begin{matrix} x \\ y \end{matrix} \right ] < \left [ \begin{matrix} -2 \\ -2 \\ 2 \\ 2 \end{matrix} \right ] z, $$

*where*

$$M = \left [ \begin{matrix} 4(2-k)+2r & (2-k)(k-3) \\ 4(2-k) & (2-k)(k-3)-2r \\ 2(k-1)+2r & (k-1)(k-2) \\ 2(k-1) & (k-1)(k-2)-2r \end{matrix} \right ]. $$

*Furthermore*, *a pattern within Hamming distance* *r* *of a* *k*-*clique converges after one iteration of the dynamics*.

#### Proof

For fixed *r* and *k*-clique **x**, there are $2^{r}$ possible patterns within Hamming distance *r* of **x**. Each of these patterns defines a pair of linear inequalities on the parameters $x,y,z$. However, only the inequalities from the following two extreme cases are active constraints. All the other inequalities are convex combinations of these.

1. *r* edges in the clique with a common node *i* are removed.
2. *r* edges are added to a node *i* not in the clique.

In the first case, there are two types of edges at risk of being mislabeled. The first are those of the form *ij* for all nodes *j* in the clique. Such an edge has $2(k-2)-r$ neighbors and ${k-2 \choose 2}$ non-neighbors. Thus, each such edge will correctly be labeled 1 after one network update if and only if *x*, *y*, and *z* satisfy

10 $$ 2(2k-r-4)x + (k-2) (k-3)y > 2z. $$

The other type are those of the form *īj* for all nodes $\bar{i} \neq i$ in the clique, and *j* not in the clique. Assuming $r < k-1$, such an edge has at most $k-1$ neighbors and ${k-1 \choose 2} - r$ non-neighbors. Thus, each such edge will be correctly labeled 0 if and only if

11 $$ 2(k-1)x + \bigl((k-1) (k-2)-2r\bigr)y < 2z. $$

Rearranging Eqs. (10) and (11) yield the first two rows of the matrix in the lemma. A similar argument applies for the second case, giving the last two inequalities.

From the derivation, it follows that if a pattern is within Hamming distance *r* of a *k*-clique, then all spurious edges are immediately deleted by case 1, all missing edges are immediately added by case 2, and thus the clique is recovered in precisely one iteration of the network dynamics. □

#### Proof of Lemma 1

The matrix inequalities in Lemma 2 define a cone in $\mathbb {R}^{3}$, and the cases $z = 1$ or $z = 0$ correspond to two separate components of this cone. For the proof of Theorem 1 in the main article, we use the cone with $z = 1$. We further assume $y = 0$ to achieve a sparsely connected matrix **W**. In this case, the second and fourth constraints are dominated by the first and third. Thus, we need *x* that solves

$$\frac{1}{2(k-1)-r} < x < \frac{1}{k-1+r}. $$

There exists such a solution if and only if

12 $$ 2(k-1)-r > k-1+r\quad \Leftrightarrow\quad k > 2r+1. $$

The above equation is feasible if and only if $r \leq\lfloor k/2 \rfloor$. □

### Proofs of Theorems 1, 2

Fix $y = 0$ and $z = 1$. We now tune *x* such that asymptotically the *α*-robustness of our set of Hopfield networks storing *k*-cliques tends to $1/2$ as $n \to\infty$. By symmetry, it is sufficient to prove robustness for one fixed *k*-clique **x**; for instance, the one with vertices $\{1, \ldots, k\}$. For $0 < p < \frac{1}{2}$, let $\mathbf{x}_{p}$ be the *p*-corruption of **x**. For each node $i \in\{1, \ldots, 2k\}$, let $i_{\mathrm{in}}, i_{\mathrm{out}}$ denote the number of edges from *i* to other clique and non-clique nodes, respectively. With an abuse of notation, we write $i \in\mathbf{x}$ to mean a vertex *i* in the clique; that is, $i \in\{1, \ldots, k\}$. We need the following inequality originally due to Bernstein from 1924.

#### Proposition 1

(Bernstein’s inequality [22])

*Let* $S_{i}$ *be independent Bernoulli random variables taking values* +1 *and* −1, *each with probability* $1/2$. *For any* $\varepsilon > 0$, *the following holds*:

$$\mathbb {P} \Biggl( \frac{1}{n} \sum_{i=1}^{n} S_{i}> \varepsilon \Biggr) \leq \exp \biggl( -\frac{n\varepsilon ^{2}}{2+2\varepsilon /3} \biggr). $$

The following fact is a fairly direct consequence of Proposition 1.

#### Lemma 3

*Let* *Y* *be an* $n \times n$ *symmetric matrix with zero diagonal*, $Y_{ij} \stackrel{\mathrm{i.i.d.}}{\sim} \operatorname{Bernoulli}(p)$. *For each* $i = 1, \ldots, n$, *let* $Y_{i} = \sum_{j}Y_{ij}$ *be the* *ith row sum*. *Let* $M_{n} = \max_{1 \leq i \leq n}Y_{i}$, *and* $m_{n} = \min_{1 \leq i \leq n} Y_{i}$. *Then*, *for any constant* $c > 0$, *as* $n \to\infty$, *we have*

$$\mathbb{P}\bigl( \vert m_{n} - np \vert > c\sqrt{n}\ln n\bigr) \to0 $$

*and*

$$\mathbb{P}\bigl( \vert M_{n} - np \vert > c\sqrt{n}\ln n\bigr) \to0. $$

*In particular*, $|m_{n} - np|, |M_{n} - np| = o(\sqrt{n}\ln n)$.

#### Proof

Fix $c > 0$. As a direct corollary of Bernstein’s inequality, for each *i* and for any $\varepsilon > 0$, we have

$$\mathbb {P}\bigl(Y_{i} - np > n\varepsilon - (p + \varepsilon )\bigr) \leq\exp \biggl( -\frac {(n-1)\varepsilon ^{2}}{2+2\varepsilon /3} \biggr). $$

It follows that

$$\mathbb {P}(Y_{i} - np > n\varepsilon ) \leq\exp \biggl( - \frac{n\varepsilon ^{2}}{4+4\varepsilon /3} \biggr), $$

and thus from a union bound with $\varepsilon = \frac{c\ln n}{\sqrt{n}}$, we have

$$\begin{aligned} \begin{aligned} \mathbb {P}\Bigl(\max_{i}Y_{i} - np > c\sqrt{n}\ln n\Bigr) &\leq \exp \biggl( -\frac {n\varepsilon ^{2}}{4+4\varepsilon /3} + \ln n \biggr) \\ & \leq \exp \biggl(-\frac{c^{2} \ln^{2} n}{4 + 4c} + \ln n \biggr). \end{aligned} \end{aligned}$$

Since this last bound converges to 0 with $n \to\infty$, we have proved the claim for $M_{n}$. Since $Y_{i}$ is symmetric about *np*, a similar inequality holds for $m_{n}$. □

#### Corollary 1

*Let* $M_{\mathrm{in}} = \max_{i \in\mathbf{x}} i_{\mathrm{in}}$, $m_{\mathrm{in}} = \min_{i \in \mathbf{x}} i_{\mathrm{in}}$, $M_{\mathrm{out}} = \max_{i \notin\mathbf{x}} i_{\mathrm{out}}$, $m_{\mathrm{out}} = \min_{i \notin\mathbf{x}} i_{\mathrm{out}}$, *and* $M_{\mathrm{between}} = \max_{i \notin\mathbf{x}} i_{\mathrm{in}}$. *Then* $M_{\mathrm{in}} - k(1-p)$, $m_{\mathrm{in}} - k(1-p)$, $M_{\mathrm{out}} - kp$, $m_{\mathrm{out}} - kp$, *and* $M_{\mathrm{between}} - kp$ *are all of order* $o(\sqrt{k}\ln k)$ *as* $k \to\infty$ *almost surely*.

#### Proofs of Theorems 1, 2 (robustness)

Let $N(e)$ be the number of neighbors of edge *e*. For each *e* in the clique:

$$N(e) \geq2m_{\mathrm{in}} + 2m_{\mathrm{out}} \sim2k + o(\sqrt{k}\ln k),\quad \mbox{w.h.p. (with high probability)}. $$

To guarantee that all edges *e* in the clique are labeled 1 after one dynamics update, we need $x > \frac{1}{N(e)}$; that is,

13 $$ x > \frac{1}{2k + o(\sqrt{k}\ln k)}. $$

If *f* is an edge with exactly one clique vertex, then we have

$$\begin{aligned} N(f) & \leq M_{\mathrm{in}} + M_{\mathrm{out}} +2M_{\mathrm{between}} \\ &\sim k(1+2p) + o(\sqrt{k}\ln k),\quad \mbox{w.h.p}. \end{aligned}$$

To guarantee that $\mathbf{x}_{f} = 0$ for all such edges *f* after one iteration of the dynamics, we need $x < \frac{1}{N(f)}$; that is,

14 $$ x < \frac{1}{k(1+2p) + o(\sqrt{k}\ln k)}. $$

In particular, if $p = p(k) \sim\frac{1}{2} - k^{\delta-1/2}$ for some small $\delta\in(0, 1/2)$, then taking $x = x(k) = \frac{1}{2} [\frac{1}{2k} + \frac{1}{k(1+2p)} ]$ would guarantee that for large *k* the two inequalities (13) and (14) are simultaneous satisfied. In this case, $\lim_{k\to \infty}p(k) = 1/2$, and thus the family of two-parameter Hopfield networks with $x(k)$, $y = 0$, $z = 1$ has robustness index $\alpha= 1/2$. □

### Clique Range Storage

In this section, we give precise conditions for the existence of a Hopfield network on $\binom{v}{2}$ nodes that stores all *k*-cliques for *k* in an interval $[m,M]$, $m \leq M \leq v$. We do not address the issue of robustness as the qualitative trade-off is clear: the more memories the network is required to store, the less robust it is. This trade-off can be analyzed by large deviation principles as in Theorem 2.

#### Lemma 4

*Fix* *m* *such that* $3 \leq m < v$. *For* $M \geq m$, *there exists a Hopfield network on* $\binom{v}{2}$ *nodes that stores all* *k*-*cliques in the range* $[m,M]$ *if and only if* *M* *solves the implicit equation* $x_{M} - x_{m} < 0$, *where*

$$\begin{aligned} x_{m} &= \frac{-(4m - \sqrt{12m^{2} - 52m + 57} - 7)}{2(m^{2} - m - 2)}, \\ x_{M} &= \frac{-(4M + \sqrt{12M^{2} - 52M + 57} - 7)}{2(M^{2}-M-2)}. \end{aligned}$$

#### Proof

Fix $z = 1/2$ and $r = 0$ in Lemma 1. (We do not impose the constraint $y = 0$.) Then the cone defined by the inequalities in Lemma 1 is in bijection with the polyhedron $\mathcal{I}_{k} \subseteq\mathbb{R}^{2}$ cut out by inequalities:

$$\begin{aligned} 4(k-2)x + (k-2) (k-3)y - 1 &> 0, \\ 2(k-1)x + (k-1) (k-2)y - 1 &< 0. \end{aligned}$$

Let $R_{k}$ be the line $4(k-2)x + (k-2)(k-3)y - 1 = 0$, and $B_{k}$ be the line $2(k-1)x + (k-1)(k-2)y - 1 = 0$. By symmetry, there exists a Hopfield network that stores all *k*-cliques in the range $[m,M]$ if and only if $\bigcap_{k=m}^{M}\mathcal{I}_{k} \neq\emptyset$. For a point $P \in\mathbb{R}^{2}$, write $x(P)$ for its *x*-coordinate. Note that, for $k \geq3$, the points $B_{k} \cap B_{k+1}$ lie on the following curve *Q* implicitly parametrized by *k*:

$$Q := \biggl\{ \biggl(\frac{1}{k-1}, \frac{-1}{(k-1)(k-2)} \biggr): k \geq3 \biggr\} . $$

When the polytope $\bigcap_{k=m}^{M}\mathcal{I}_{k}$ is nonempty, its vertices are the following points: $R_{M} \cap R_{m}$, $R_{M} \cap B_{m}$, $B_{k} \cap B_{k+1}$ for $m \leq k \leq M-1$, and the points $B_{M} \cap R_{m}$. This defines a nonempty convex polytope if and only if

$$x_{M} := x(Q \cap R_{M}) < x_{m} := x(Q \cap R_{m}). $$

**Fig. 6:**
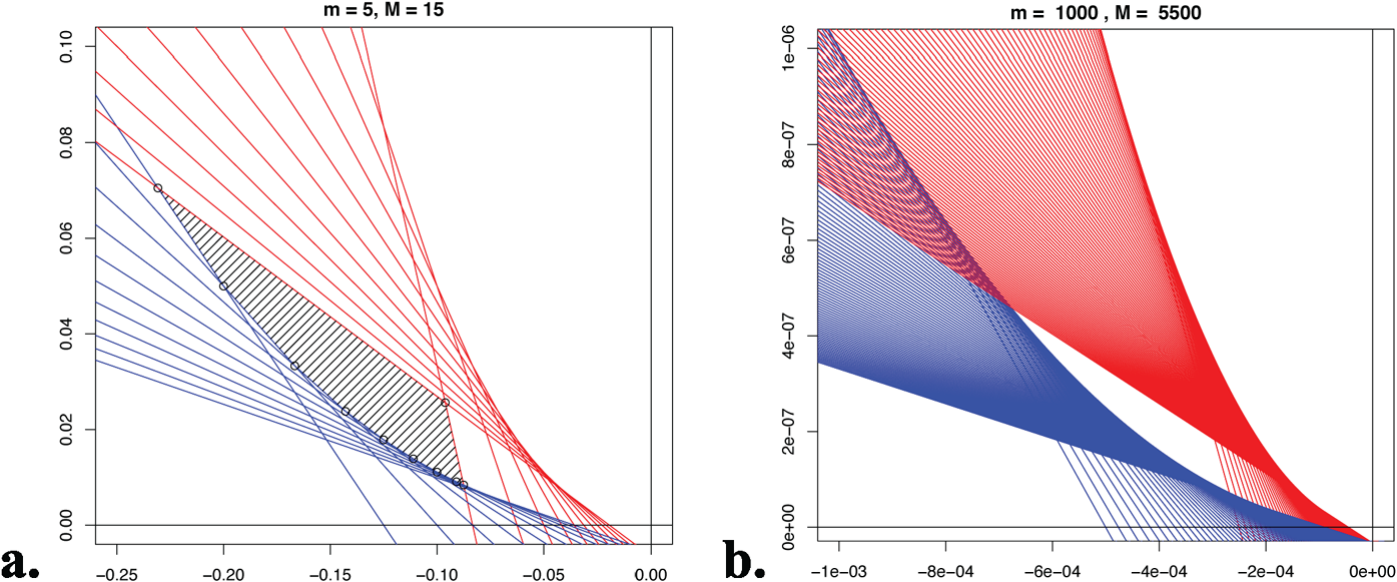
Feasible exponential storage. **(a)** The shaded region is the feasible polytope for network parameters giving clique storage for $5 \leq k \leq15$. *Black points* are its vertices, *the red* $R_{k}$ and *blue* $B_{k}$ *lines* are linear constraints. **(b)** Lines $R_{k}$ (*red*) and $B_{k}$ (*blue*) for $1000 \leq k \leq5500$. Note the appearance of the smooth curve *Q* enveloping the family $B_{k}$ in the figure

Direct computation gives the formulas for $x_{m}$, $x_{M}$ in the lemma statement. See Fig. 6 for a visualization of the constraints of the feasible region.

□

Fixing the number of nodes and optimizing the range $M - m$ in Lemma 4, we obtain Theorem 3 from Sect. 3.

#### Proof of Theorem 3

From Lemma 4, for large *m*, *M*, and *v*, we have the approximations $x_{m} \approx\frac{\sqrt{12}-4}{2m}$, $x_{M} \approx\frac {-\sqrt{12}-4}{2M}$. Hence $x_{M} - x_{m} < 0$ when $M \lesssim\frac {2+\sqrt{3}}{2-\sqrt{3}}m = Dm$. Asymptotically for large *v*, the most cliques are stored when $M = Dm$ and $[m,M]$ contains $v/2$. Consider $m = \beta v$ so that $v \geq M = D\beta v \geq v/2$, and thus $1/D \geq\beta\geq1/(2D)$. Next, set $u = v/2 - m = v(1/2-\beta)$ and $w = M - v/2 = v(D\beta- 1/2)$ so that storing the most cliques becomes the problem of maximizing over admissible *β* the quantity:

$$\max\{u,w\} = \max\bigl\{ v(1/2-\beta),v(D\beta-1/2)\bigr\} . $$

One can now check that $\beta= 1/D$ gives the best value, producing the range in the statement of the theorem.

Next, note that $\binom{v}{k}2^{-v}$ is the fraction of *k*-cliques in all cliques on *v* vertices, which is also the probability of a $\operatorname{Binom}(v, 1/2)$ variable equaling *k*. For large *v*, approximating this variable with a normal distribution and then using Mill’s ratio to bound its tail c.d.f. *Φ*, we see that the proportion of cliques storable tends to

$$1 - \varPhi \biggl(\frac{D-1}{D} \sqrt{v} \biggr) \approx1 - \exp(-Cv), $$

for some constant $C \approx\frac{(D-1)^{2}}{2D^{2}} \approx0.43$. □

### Hopfield–Platt Networks

We prove the claim in the main text that Hopfield–Platt networks [13] storing all permutations on $\{1,\ldots,k\}$ will not robustly store *derangements* (permutations without fixed-points). For large *k*, the fraction of permutations that are derangements is known to be $e^{-1} \approx0.36$.

#### Proof of Theorem 4

Fix a derangement *σ* on $\{1,\ldots,k\}$, represented as a binary vector **x** in $\{0,1\}^{n}$ for $n = k(k-1)$. For each ordered pair $(i,j)$, $i \neq j$, $j \neq\sigma(i)$, we construct a pattern $\mathbf{y}_{ij}$ that differs from **x** by exactly two bit flips:

1. Add the edge *ij*.
2. Remove the edge $i\sigma(i)$.

There are $k(k-2)$ such pairs $(i,j)$, and thus $k(k-2)$ different patterns $\mathbf{y}_{ij}$. For each such pattern, we flip two more bits to obtain a new permutation $\mathbf{x}^{ij}$ as follows:

1. Remove the edge $\sigma^{-1}(j)j$.
2. Add the edge $\sigma^{-1}(j)\sigma(i)$.

It is easy to see that $\mathbf{x}^{ij}$ is a permutation on *k* letters with exactly two cycles determined by $(i,j)$. Call the set of edges modified the *critical edges* of the pair $(i,j)$. Note that $\mathbf{x}^{ij}$ are all distinct and have disjoint critical edges.

Each $\mathbf{y}_{ij}$ is exactly two bit flips away from **x** and $\mathbf{x}^{ij}$, both permutations on *k* letters. Starting from $\mathbf{y}_{ij}$, there is no binary Hopfield network storing all permutations that always correctly recovers the original state. In other words, for a binary Hopfield network, $\mathbf{y}_{ij}$ is an *indistinguishable* realization of a corrupted version of **x** and $\mathbf{x}^{ij}$.

We now prove that, for each derangement **x**, with probability at least $1 - (1-4p^{2})^{n/2}$, its *p*-corruption $\mathbf{x}_{p}$ is indistinguishable from the *p*-corruption of some other permutation. This implies the statement in the theorem.

For each pair $(i,j)$ as above, recall that $\mathbf{x}_{p}$ and $\mathbf {x}^{ij}_{p}$ are two random variables in $\{0,1\}^{n}$ obtained by flipping each edge of **x** (resp. $\mathbf{x}^{ij}$) independently with probability *p*. We construct a coupling between them as follows. Define the random variable $\mathbf{x}'_{p}$ via:

- For each non-critical edge, flip this edge on $\mathbf{x}'_{p}$ and $\mathbf{x}^{ij}$ with the same $\operatorname{Bernoulli}(p)$.
- For each critical edge, flip them on $\mathbf{x}'_{p}$ and $\mathbf {x}^{ij}$ with independent $\operatorname{Bernoulli}(p)$.

Then $\mathbf{x}'_{p} \stackrel{d}{=} \mathbf{x}_{p}$ have the same distribution, and $\mathbf{x}'_{p}$ and $\mathbf{x}^{ij}_{p}$ only differ in distribution on the four critical edges. Their marginal distributions on these four edges are two discrete variables on 2^4^ states, with total variation distance $1 - 4(1-p)^{2}p^{2}$. Thus, there exists a random variable $\mathbf{x}''_{p}$ such that $\mathbf{x}''_{p} \stackrel{d}{=} \mathbf{x}'_{p} \stackrel{d}{=} \mathbf{x}_{p}$, and

$$\mathbb{P}\bigl(\mathbf{x}''_{p} = \mathbf{x}^{ij}_{p}\bigr) = 4(1-p)^{2}p^{2}. $$

In other words, given a realization of $\mathbf{x}^{ij}_{p}$, with probability $4(1-p)^{2}p^{2}$, this is equal to a realization from the distribution of $\mathbf{x}_{p}$, and therefore no binary Hopfield network storing both $\mathbf{x}^{ij}$ and **x** can correctly recover the original state from such an input. An indistinguishable realization occurs when two of the four critical edges are flipped in a certain combination. For fixed **x**, there are $k(k-2)$ such $\mathbf{x}^{ij}$ where the critical edges are disjoint. Thus, the probability of $\mathbf{x}_{p}$ being an indistinguishable realization from a realization of one of the $\mathbf{x}^{ij}$ is at least

$$1 - \bigl(1 - 4(1-p)^{2}p^{2}\bigr)^{k(k-2)} > 1 - \bigl(1-4p^{2}\bigr)^{n/2}, $$

completing the proof of Theorem 4. □

### Examples of Clique Storage

**Fig. 7:**
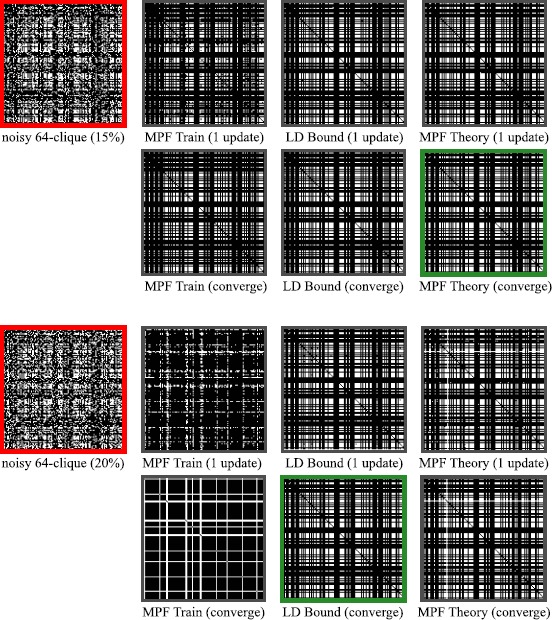
Examples of robustness for networks in Fig. 4 of main text with $v = 128$, $k = 64$, $n = 8128$. Adjacency matrices of noisy cliques (in *red*) have 1219 (*top*) or 1625 (*bottom*) bits corrupted out of 8128 ($p=0.15 / 0.2$) from the original 64-clique (in *green*). Images show the result of dynamics applied to these noisy patterns using networks with all-to-all MPF parameters after L-BFGS training on $50\text{,}000$ 64-cliques (${\approx}2\mathrm{e}{-}31\%$ of all 64-cliques), Large deviation parameters $(x, y, z) = (0.0091, 0, 1)$, or MPF Theory parameters $(x, y, z) = (0.0107, 0, 1)$ from Eq. (7) in the main text

To illustrate the effect of two different noise levels on hidden clique finding performance of the networks from Fig. 4, we present examples in Fig. 7 of multiple networks acting with their dynamics on the same two noisy inputs. Notice that non-clique fixed-points appear, and it is natural to ask whether a complete characterization of the fixed-point landscape is possible. Intuitively, our network performs a local, weighted degree count at each edge of the underlying graph and attempts to remove edges with too few neighbors, while adding in edges that connect nodes with high degrees. Thus, resulting fixed-points (of the dynamics) end up being graphs such as cliques and stars. Beyond this intuition, however, we do not have a way to characterize all fixed-points of our network in general.

In fact, this is a very difficult problem in discrete geometry, and except for toy networks, we believe that this has never been done. Geometrically, the set of all states of a binary Hopfield network with *n* neurons is the *n*-hypercube $\{0,1\}^{n}$. Being a fixed-point can be characterized by the energy function becoming larger when one bit is flipped. As the energy function is quadratic, for each of the *n* bits flipped, this creates a quadratic inequality. Thus, the set of all fixed-point attractors in a binary Hopfield network is the *n*-hypercube intersected with *n* quadratic inequalities in *n* variables. In theory, one could enumerate such sets for small *n*; however, characterizing them all is challenging, even for the highly symmetric family of weight matrices that we propose here.

## Abbreviations

OPR: outer-product rule
MPF: minimum probability flow
